# Identification of Immune Responses to Japanese Encephalitis Virus Specific T Cell Epitopes

**DOI:** 10.3389/fpubh.2020.00019

**Published:** 2020-02-12

**Authors:** Pradeep Darshana Pushpakumara, Chandima Jeewandara, Ayesha Wijesinghe, Laksiri Gomes, Graham S. Ogg, Charitha Lakshini Goonasekara, Gathsaurie Neelika Malavige

**Affiliations:** ^1^Department of Preclinical Sciences, Faculty of Medicine, General Sir John Kotelawala Defence University, Rathmalana, Sri Lanka; ^2^Centre for Dengue Research, University of Sri Jayewardenepura, Nugegoda, Sri Lanka; ^3^MRC Human Immunology Unit, Weatherall Institute of Molecular Medicine, University of Oxford, Oxford, United Kingdom

**Keywords:** Japanese encephalitis virus, dengue virus, cross reactive T cell responses, cultured ELISpot assays, highly conserved regions

## Abstract

**Background:** Due to the similarity between the dengue (DENV) and the Japanese encephalitis virus (JEV) there is potential for immune cross-reaction. We sought to identify T cell epitopes that are specific to JEV and do not cross react with DENV.

**Methodology:** 20mer peptides were synthesized from regions which showed >90% conservation. Using IFNγ cultured ELISpot assays, we investigated JEV-specific T cell responses in DENV^−^ and JEV^−^ non-immune individuals (DENV^−^JEV^−^ = 21), JEV seronegative and had not received the JE vaccine, but who were DENV seropositive (DENV^+^JEV^−^ = 22), JEV^+^(seropositive for JEV and had received the JE vaccine), but seronegative for DENV (DENV^−^JEV^+^ = 23). We further assessed the responses to these peptides by undertaking *ex vivo* IFNγ assays and flow cytometry.

**Results:** None of DENV^−^JEV^−^ individuals responded to any of the 20 JEV-specific peptides. High frequency of responses was seen to 6/20 peptides by individuals who were JEV^+^ but DENV^−^, where over 75% of the individuals responded to at least one peptide. P34 was the most immunogenic peptide, recognized by 20/23 (86.9%) individuals who were DENV^−^JEV^+^, followed by peptide 3 and peptide 7 recognized by 19/23 (82.6%). Peptide 34 from the NS2a region, showed <25% homology with any flaviviruses, and <20% homology with any DENV serotype. Peptide 20 and 32, which were also from the non-structural protein regions, showed <25% homology with DENV. *Ex vivo* responses to these peptides were less frequent, with only 40% of individuals responding to peptide 34 and 16–28% to other peptides, probably as 5/6 peptides were recognized by CD4+ T cells.

**Discussion:** We identified six highly conserved, T cell epitopes which are highly specific for JEV, in the Sri Lankan population. Since both JEV and DENV co-circulate in the same regions and since both JE and dengue vaccines are likely to be co-administered in the same geographical regions in future, these JEV-specific T cell epitopes would be useful to study JEV-specific T cell responses, in order to further understand how DENV and JEV-specific cellular immune responses influence each other.

## Introduction

Mosquito borne viral infections are one of the leading emerging infectious diseases and represent a major public health problem in many tropical and subtropical countries. Among the rapidly emerging flaviviruses, infections due to the dengue viruses (DENV) are the most common, with the incidence increasing from 285.3 per 100,000 individuals in 1990–1371.1 in 2013 (1). Although case fatality rates due to dengue are declining in many countries including Sri Lanka, the rates are still significantly high in countries such as India, where the case fatality rates are estimated to be 2.6% (2). Other flavivirus infections such as the Japanese Encephalitis virus (JEV) and the West Nile virus (WNV), co-circulate in the same geographical regions such as DENV (3, 4), and due to the similarity between these viruses, have a potential to modulate the immune responses to each other. Natural infection with JEV has shown to generate highly cross reactive T cell responses that has a potential to lead to either milder or more severe disease when infected with DENV (5).

The studies which describe the effect of pre-existing JEV immunity on the outcome of DENV infection have shown varied results. A large prospective study carried out in Thailand showed that individuals with neutralizing antibodies to JEV, had a significantly increased risk of developing symptomatic dengue (6). In contrast another study in Thailand showed that those who received the inactivated JEV vaccine were less likely to get severe dengue (7). In a previous study, we observed that those who were seropositive for JEV were more likely to have been hospitalized due to dengue, compared to those who were seronegative for JEV (8). However, due to the cross-reactive nature of DENV-specific antibodies with JEV, it could not be ascertained if JEV positivity was due to the presence of highly cross reactive DENV-specific antibodies, or due to actual infection with JEV. Therefore, currently it is still not clear if DENV or JEV-specific antibody and T cell responses influence the immune responses to each other virus during subsequent infection and thus influence the disease outcome.

Both CD8+ and CD4+ T cells have been shown to play an important role in protection against DENV, JEV and Zika virus (5, 9–12). Individuals who were naturally exposed to JEV were shown to have antibody and T cell responses, that showed high cross-reactivity with DENV (5, 13). In our previous studies we showed that T cell responses of 20–30% of individuals who were naturally infected with DENV were cross-reactive with JEV (14). Apart from the magnitude of the T cell response, the functionality of T cell responses, specific to either JEV or DENV, have been shown to associate with the clinical disease outcome in both infections (5, 15). Virus-specific T cells of patients who had a milder clinical disease (either JEV or DENV), had different polyfunctional T cell signatures compared to those who had more severe disease (5, 12, 15). Due to the similarity of JEV and DENV, infection or immunization with either virus has a potential to influence both the magnitude and the functionality of T cell responses to each other. Although flavivirus cross-reactive T cells are likely to be cross protective, it is difficult to speculate on such protection in the absence of data regarding either reduced or enhanced disease severity following sequential infection with flaviviruses.

The occurrence of mild/asymptomatic illness in the majority of DENV infected individuals, and severe dengue and death in some individuals, has been attributed to many risk factors such as a secondary dengue infection, the time interval between two dengue infections (16), the incidence of dengue infection in a particular year and preceding years (17) and the presence of co-morbid illnesses (18, 19). Although disease enhancement due to the presence of non-neutralizing antibodies and possibly cross-reactive T cells is thought to lead to severe disease (20), DHF and fatalities have also been reported in primary dengue infection in the absence of DENV specific antibodies or T cells (21, 22). The presence of T cell responses that cross-react with other flaviviruses such as JEV, has a potential to be protective or to be involved in disease pathogenesis leading to severe clinical disease when individuals are naturally infected with DENV and have the potential to modulate immune responses to dengue vaccines (23). As the incidence of dengue and other flaviviruses are on the rise and as several dengue vaccine candidates are currently undergoing clinical trials, it would be important to investigate how the immune response to one of these co-circulating flaviviruses, influence the disease outcome during subsequent infections with other flaviviruses.

In order to determine if JEV-specific T cell responses are indeed cross protective when infected with DENV, it would be initially important to differentiate JEV-specific T cell responses from those which are broadly cross-reactive with DENV. This would be important especially in order to understand how sequential infection with different flaviviruses or immune responses induced by vaccination against JEV, would subsequently influence the disease outcome when naturally infected or vaccinated with DENV. It was recently shown that infection with JEV was far commoner than previously thought in DENV endemic countries and interpretation of natural infection with JEV was difficult especially following secondary dengue infections, due to the presence of more cross-reactive heterotypic antibodies (13). Therefore, as an initial step it would be important to identify T cell epitopes that are specific to JEV and do not cross-react with DENV in order to identify individuals who have had natural JEV infection, and also to further investigate T cell responses to JEV, independent of DENV-specific T cell responses. In this study, we identified JEV-specific, DENV non cross-reactive T cell epitopes and we proceeded to determine the immunogenetic JEV-specific T cell responses both *ex vivo* and by cultured ELISpot assays in individuals who received JEV vaccine and those who were naturally infected with DENV but were non-immune to JEV.

## Materials and Methods

### Identification of JEV-Specific Highly Conserved Regions Within JEV

One hundred and twelve JEV polyprotein sequences, which were isolated within a period of 50 years from the South Asian and South East Asian regions were retrieved from National Center for Biotechnology information. These sequences were aligned using ClustalW, on Mega 7 software (www.megasoftware.net/) to identify the degree of conservation. Regions which showed >90% conservation were identified and sectioned into 20mer peptides overlapping by 5 or 10 amino acids. The specificity of these JEV peptides was determined by using Clustal Omega of European Bioinformatics Institute (EBI) (www.ebi.ac.uk) to confirm that they did not significantly cross-react with DENV (Supplementary Table 1). Out of these 36 peptides, only 20 peptides were successful in the synthesis with 90% purity (GENEscript USA) and were used for further analysis.

### Recruitment of Healthy Individuals to Identify JEV Peptide Specific T Cell Responses

In order to identify the immunogenic JEV peptides from the 20 JEV-specific peptides identified above, we recruited 66 individuals, through the Family Practice Center, University of Sri Jayewardenepura, which is the primary health care facility of the University. These 66 individuals comprised of 21 individuals who were seronegative for both JEV and DENV, 22 were seronegative for JEV (and had not received the JE vaccine) but were DENV seropositive, 23 individuals were seronegative for DENV and had received the JE vaccine (Table 1). These individuals were initially recruited in year 2013 as a part of a large longitudinal community cohort study (*n* = 1,689) (14) and were invited to provide an additional sample of blood in year 2018, to re-evaluate their serostatus to DENV and JEV. In order to re-evaluate their serostatus at the time of donating a blood sample to this study, a serum sample was also obtained for detection of JEV and DENV IgG at the time of obtaining PBMCs (see below for details regarding the assay). These cohort of individuals have been followed by us through 2013, and all cases of febrile episodes for reported to the Family Practice Center.

**Table 1:** Number of individuals recruited for culture and *ex vivo* ELISpot assays.

**Group**	**Number of individuals**
**RECRUITED INDIVIDUALS FOR CULTURED ELISpot ASSAYS**	
DENV^−^JEV^−^	21
DENV^+^JEV^−^	22
DENV^−^JEV^+^ (JE vaccinated)	23
Total	66
**RECRUITED INDIVIDUALS FOR** ***EX VIVO*** **ELISpot ASSAYS**	
DENV^−^JEV^−^	20
DENV^+^JEV^−^	25
DENV^−^JEV^+^ (JE vaccinated)	25
DENV^+^JEV^+^ (JE vaccinated)	25
Total	95
Total number of recruits	161

Due to the limitations of the PBMC samples of the above 66 individuals, we recruited an additional cohort of 95 individuals for further assessment of the *ex vivo* IFNγ ELISpot responses to JEV-specific, immunodomoninant peptides. These 95 individuals too were initially recruited in 2013 as a part of the large community study (14). However, as 6 years had elapsed since 2013, we re-evaluated their serostatus for JEV and DENV IgG at the time of obtaining blood samples in 2019 for *ex vivo* ELISpot assays. PBMCs were extracted from these fresh blood samples and the *ex vivo* ELISpot assays we carried out using the fresh PBMCs. The time elapsed between JEV vaccination in these two cohorts of individuals was a mean of 17.15 years (SD ± 2.4 years).

Of the 95 individuals recruited for the *ex vivo* ELISpot assays, 20 were seronegative for JEV and DENV (DENV^−^JEV^−^), 25 were seropositive for DENV and were seronegative for JEV (and had not received the JE vaccine) (DENV^+^JEV^−^), 25 were seropositive for JEV (and had received the JE vaccine), but seronegative for DENV (DENV^−^JEV^−^), 25 were seropositive for both JEV and DENV (DENV^+^JEV^+^) (Table 1). Fresh PBMC samples were used for both culture and *ex vivo* ELISpot assays.

Ethical approval for this study was granted by Ethics Review Committee of the University of Sri Jayewardenepura.

### Determining DENV and JEV Serostatus in Healthy Individuals

The seropositivity of individuals to DENV was assessed using the indirect dengue IgG capture ELISA (Panbio, Australia) (8) and for JEV by JE direct IgG ELISA (InBios International, USA). Immune status to JEV was calculated using the immune status ratio (ISR) according to the manufacturers' instructions. An ISR of >5 was considered positive; an ISR of 2–5 equivocal and an ISR of <2 was considered negative.

Of these individuals, those who had not received the JE vaccine and were also seronegative for JEV IgG antibodies by a commercial ELISA (Inbios, USA), were considered as JEV seronegative (JEV^−^). Those who had received the JE vaccine and who had detectable JEV IgG antibodies by a commercial ELISA were considered as JEV seropositive (JEV^+^). Individuals who had received the JE vaccine and were seronegative by the commercial JEV IgG ELISA or those who had not received the JE vaccine and were seropositive based on the commercial JEV IgG ELISA were not considered in the analysis. DENV seropositivity of these individuals were identified by using commercially available dengue IgG panbio ELISA kit (Australia).

### Cultured ELISpot Assays

Cultured ELISpot assays were performed to identify JEV-specific peptides recognized by memory T cells of JEV immune individuals, as previously described (14, 24). Cultured ELISpot assays have been previously used to detect antigen specific memory T cells, especially present in low frequency in HIV infection, Epstein Barr virus infection, malaria, hepatitis C infection and memory T cell responses to the DENV in acute dengue and in healthy DENV seropositive individuals (14, 25–27).

The responses to these 20 JEV-specific peptides were assessed in DENV and JEV seronegative individuals (DENV^−^JEV^−^, *n* = 21), DENV seronegative individuals who were vaccinated for JEV (DENV^−^ JEV^+^, *n* = 23), and DENV seropositive individuals who were not vaccinated for JEV (DENV^+^ JEV^−^, *n* = 22). Briefly, 5.0 × 10^6^ PBMCs were incubated for 10 days with 20 μl of the JE vaccine (SA 14-14-2 live attenuated) in a 24 well plate. The SA 14-14-2 is a mouse brain derived, live attenuated JE vaccine has been attenuated for neurovirulence with changes in 57 nucleotides resulting in changes in 24 amino acids compared to the live virus (28). IL-2 was added on day 3 and 7 at a concentration of 100 units/ml. All cell lines were routinely maintained in RPMI 1,640 supplemented with 2 mM L-glutamine, 100 IU/ml penicillin and 100 μg/ml plus 10% human serum at 37°C, in 5% CO_2._ T cell lines were tested individually after 10 days culture for responses to the 20 JEV-specific 20mer peptides. Briefly, ELISpot plates (Millipore Corp., Bedford, USA) were coated with anti-human IFNγ antibody overnight (Mabtech, Sweden). For cultured ELISpot assays, 4 × 10^5^ cultures cells were added to a final volume of 200 μl. JEV-specific peptides were added at a final concentration of 10 μM as previously described (29, 30). All peptides were tested in duplicate. PHA was always included as a positive control and media alone with the cells alone was included as a negative control. The plates were incubated overnight at 37°C and 5% CO_2_. The cells were removed, and the plates developed with a second biotinylated Ab to human IFNγ and washed a further six times. The plates were developed with streptavidin-alkaline phosphatase (Mabtech AB) and colorimetric substrate, and the spots enumerated using an automated ELISpot reader. Background (cells plus media) was subtracted and data expressed as number of spot-forming units (SFU) per 10^6^ PBMC.

### *Ex vivo* ELISpot Assays

As the cultured ELISpot responses predominantly assess central memory T cells, in order to assess the *ex vivo* effector memory T cell responses to these peptides, we assessed *ex vivo* IFNγ ELISpot responses in 95 individuals to 6/20 peptides, which were identified as being immunogenic with the cultured ELISpot assays (P2, P3, P7, P20, P32, and P34). The *ex vivo* IFNγ ELISpot responses were assessed in DENV and JEV seronegative individuals (DENV^−^JEV^−^, *n* = 20), DENV seronegative individuals who were vaccinated for JEV (DENV^−^JEV^+^, *n* = 25), DENV seropositive individuals who were not vaccinated for JEV (DENV^+^ JEV^−^, *n* = 25), and DENV seropositive individuals who were vaccinated for JEV (DENV^+^ JEV^+^, *n* = 25).

*Ex vivo* ELISpot assays were performed as previously described [see detailed description under cultured ELISpot assays (14, 31, 32)]. In *ex vivo* ELISpot assays PBMCs 1 × 10^5^ were added to each well and JEV-specific, conserved peptides were added at a final concentration of 10 μM as previously described and tested in duplicate (32) The spots were enumerated using an automated ELISpot reader (AID, Germany). Background (cells with media) was subtracted and data expressed as number of spot-forming units (SFU) per 10^6^ PBMC. All peptides that induced an IFN-γ response of more than mean ± 3 standard deviations of the negative controls were considered positive.

### Flow Cytometry

To identify the subtype of T cells that were responding to JEV specific peptides, intracellular cytokine staining of PBMCs were performed. As we assessed the IFNγ production by *ex vivo* and cultured ELISpot assays, we assessed the degranulation capacity of JEV-specific T cells by carrying out CD107a expression in responses to these peptides *ex vivo*.

Briefly, the PBMCs were stimulated at 2 × 10^6^/ml in RPMI-1640 plus 10% heat inactivated human serum with the relevant peptides (10 μM) for 16 h according to the manufacturer's instructions in the presence of Monensin (2 μM) (Biolegend, USA). The following monoclonal antibodies from Biolegend, USA, were used in this study after optimization by serial dilutions: anti CD3-APC Cy7 (clone OKT3), anti CD8-PE™ (clone SK1), anti CD4 Pacific blue (clone OKT4), CD107a FITC (clone H4A3) and LIVE/DEAD Fixable Aqua Dead Cell Stain Kit were used. Intracellular staining was carried out as previously described (27, 33). To determine CD107a expression, PBMCs were stained with anti CD107a-FITC monoclonal antibodies for 30 min at 1–2 × 10^6^/ml in RPMI 1640 plus 10% FCS, prior to stimulation with the antigen (15). PBMCs were stained with anti CD3, anti CD4 and CD8, permeabilized and fixed with Cytofix/Cytoperm (Biolegend, USA) and acquired using a Guava-easy Cyte 12 HT Flowcytometer (Merck, Germany) and analyzed with FCS express 6 Flow Research Edition. A hierarchical gating strategy was used to gate live, single, CD3+, CD4+, and CD8+ T cells. Each antibody was titrated to determine the optimum concentration to use by comparing it with Fluorescence Minus One (FMO) controls.

### Statistical Analysis

PRISM version 8.1 was used in statistical analysis, which was used to analyse the responses to individual JEV peptides. As the data were not normally distributed, differences in means were compared using the Mann–Whitney *U*-test (two tailed).

## Results

### Identification and Specificity of JEV Peptides

Using bioinformatic tools, although we identified 36 JEVspecific, highly conserved regions within JEV, only 20/36 20mer JEV specific peptides representing these regions were successfully synthesized. The region within JEV where these peptides were identified and the homology of these regions with other flaviviruses and the 4 DENV serotypes is shown in Table 2. Although the structural proteins represent <20% of the whole JEV polyprotein, the 14/20 JEV-specific peptides were identified within the structural regions and 11/14 peptides within the envelope region. Only 6/20 identified JEV-specific peptides were located within the regions representing the non-structural proteins. The SA 14-14-2, live attenuated JE vaccine which was used as the antigen to stimulate PBMCs in this study, has changes in 57 nucleotides resulting in changes in 24 amino acids. These changes in the amino acids between the wild type virus and the JE vaccine virus was only seen in peptide 11. None of the other peptides, were within the regions where the changes in the amino acids were seen between the wild type viruses and the vaccine virus.

**Table 2:** The homology of JEV-specific peptides with four dengue serotypes, WNV, YFV, and Zika virus.

**No**.	**Peptide sequence**	**Protein**	**Peptide ID**	**DENV1%**	**DENV2%**	**DENV3%**	**DENV4%**	**WNV%**	**YFV%**	**Zika%**
1	^20^GLPRVFPLVGVKRVVMSLLDG^39^	Capsid	P1	30	25	30	30	55	50	40
2	^155^YSAQVGASQAAKFTVTPNAP^174^	Envelope	P2	30	15	35	15	55	20	25
3	^149^SENHGNYSAQVGASQAAKFT^168^	Envelope	P3	25	25	25	25	55	35	25
4	^331^SDGPCKIPIVSVASLNDMTP^350^	Envelope	P5	35	30	30	25	75	35	35
5	^341^SVASLNDMTPVGRLVTVNPF^360^	Envelope	P6	35	35	25	25	90	35	40
6	^351^VGRLVTVNPFVATSSANSKV^370^	Envelope	P7	30	35	35	30	75	30	40
7	^53^LAEVRSYCYHASVTDISTVA^72^	Envelope	P8	20	30	25	35	70	30	45
8	^77^TGEAHNKKRADSSYVCKQG^95^	Envelope	P9	30	25	35	25	60	30	40
9	^194^SGLNTEAFYVMTVGSKSFLV^213^	Envelope	P10	25	20	25	25	65	30	35
10	^261^GLHQALAGAIVVEYSSSVKL^280^	Envelope	P11	30	35	25	30	75	35	30
11	^471^MGVNARDRSIALAFLATGGV^490^	Envelope	P12	30	25	20	35	80	15	45
12	^481^ALAFLATGGVLVFLATNVHA^500^	Envelope	P13	25	25	25	35	70	20	50
13	^121^LQIGVHGILNAAAIAWMIVR^140^	NS2A	P14	15	15	15	20	40	25	25
14	^108^NESSIMWLASLAIVTACAG^126^	Capsid	P16	15	15	15	20	20	30	25
15	^73^SSQAGSLFVLPRGVPFTDLD^92^	NS4B	P18	30	30	35	30	50	25	40
16	^11^ADLKSMFAGKTQASGLTGLP^33^	NS4B	P19	20	20	20	20	40	25	30
17	^21^TQASGLTGLPSMALDLRPAT^40^	NS4B	P20	20	25	20	20	50	30	35
18	^100^KQNKRGGNEGSIMWLACLAV^119^	Capsid	P32	10	15	10	25	40	25	25
19	^105^QITLTTFLTAMVLATLHYGY^124^	NS4B	P33	25	25	25	30	60	25	35
20	^111^AAFFQLASADLQIGVHGILN^130^	NS2A	P34	20	10	10	20	40	25	25

*Protein sequence in the table showed as ‘N' terminal to ‘C' terminal, and superscript number showed the peptide position in the relevant protein*.

### Identification of JEV Specific Immunogenic Peptides in JEV Immune Individuals Through Cultured ELISpot Assays

**Figure 1:**
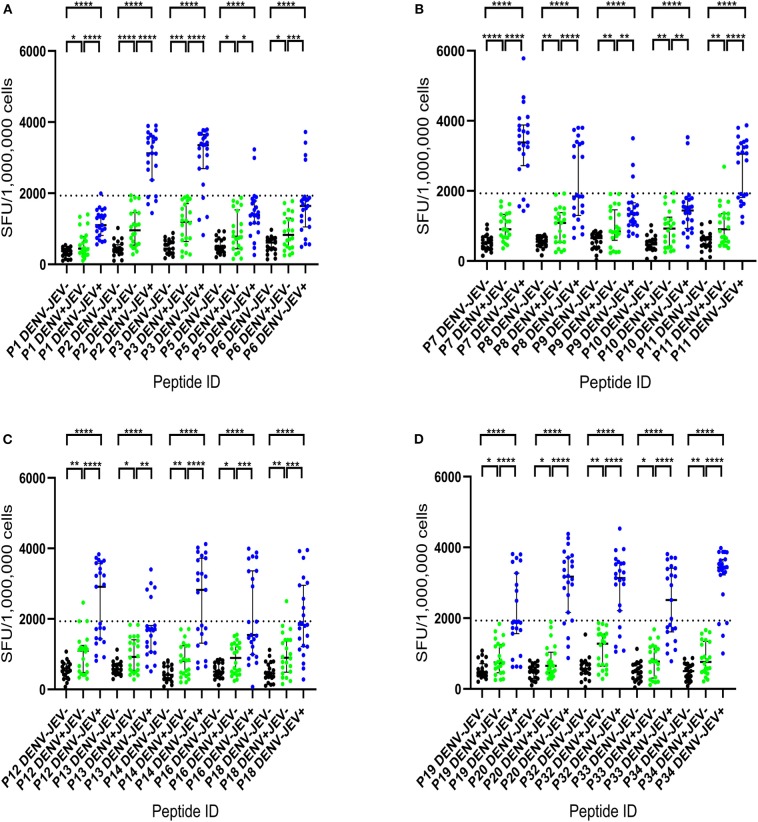
IFNγ cultured ELISpot responses to 20 JEV-specific peptides in individuals with varied DENV and JEV seropositivity. T cell responses to twenty 20mer JEV-specific peptides were measured by following short term culture with IFNγ ELISpot in those who were JEV seropositive but seronegative for DENV (JEV^+^DEV^−^, *n* = 23), DENV seropositive but JEV seronegative (JEV^−^DENV^+^, *n* = 22) and seronegative for both (JEV^−^DENV^−^, *n* = 21). Error bars indicate the median and the interquartile range. The horizontal dotted line represents the cut-off value of 1,930 SFU/10^6^, which was considered as the mean, ± 3SD of the negative controls given spot count for all three groups. ^*^P < 0.05, ^**^P < 0.01, ^***^P < 0.001, ^****^P < 0.0001. The background has been subtracted from the responses displayed. Black color- JEV^−^DENV^−^; Green color- JEV^−^DENV^+^; Blue color- JEV^+^DEV^−^. **(A)** IFNγ cultured ELISpot responses of P1 to P6. **(B)** IFNγ cultured ELISpot responses of P7 to P11. **(C)** IFNγ cultured ELISpot responses of P12 to P18. **(D)** IFNγ cultured ELISpot responses of P19 to P34.

Cultured ELISpot assays have widely used as a sensitive assay that measures central memory T cells that are even present at low frequency (25, 34, 35). Therefore, we used this approach to identify JEV-specific memory T cell responses, that could be even be present low frequency and therefore, be missed by using *ex vivo* ELISpot assays. Cultured ELISpot responses to the 20 JEV-specific 20mer peptides in the JE vaccinated, DENV seronegative individuals (DENV^−^JEV^+^, *n* = 23), DENV seropositive but JEV non-vaccinated individuals (DENV^+^ JEV^−^, *n* = 22), and both DENV and JEV seronegative individuals (DENV^−^ JEV^−^, *n* = 21) are shown in Figure 1. Cutoff value for a positive T cell response was considered as the mean ± 3SD of all negative controls, and in this study, it was ± 1,930 SFU/10^6^ PBMCs. Although quite a few responses were considered negative based on these criteria, we wished to have a more stringent assessment criteria to only select the T cell responses which displayed a high magnitude. This was to avoid selection of any responses that would be false positive. An example of a plate layout and responses to the JEV specific peptides, the negative and positive control is shown in Supplementary Figure 1.

None of JEV and DENV seronegative individuals (DENV^−^JEV^−^) responded to any of these peptides, while three individuals who were DENV seropositive but JEV seronegative responded to P11, P12, and P18 (Figure 1). Responses to other JEV-specific peptides were not detected in any of DENV^+^JEV^−^ individuals. P34 was the most immunogenic JEV-specific peptide, recognized by 20/23 (86.9%) individuals who were DENV^−^JEV^+^ (Figure 1). P3 and P7 were recognized by 19/23 (82.6%) of DENV^−^JEV^+^ group of individuals and P2, P32, and P20 recognized by 18/23 (78.3%) individuals (Table 3). The alignment of each of these peptides with DENV, ZIKV, YFV and WNV is shown in Supplementary Figure 2. Only 14 (61%) of individuals responded to peptide 11 (aa261–280), where there is a one amino acid difference between the vaccine virus and the wild type JEV.

**Table 3:** IFNγ cultured ELISpot responses to JEV-specific peptides.

**Peptide ID**	**Peptide sequence**	**Protein**	**Number of individuals who responded (*n* = 23)**	**Median (IQR) SFU/10^**6**^ cells**
P1	^20^GLPRVFPLVGVKRVVMSLLDG^39^	Capsid	1 (4%)	1,100 (810–1,390)
P2	^155^YSAQVGASQAAKFTVTPNAP^174^	Envelope	18 (78%)	3,120 (2,370–3,580)
P3	^149^SENHGNYSAQVGASQAAKFT^168^	Envelope	19 (83%)	3,350 (2,690–3,650)
P5	^331^SDGPCKIPIVSVASLNDMTP^350^	Envelope	2 (9%)	1,380 (1,140–1,780)
P6	^341^SVASLNDMTPVGRLVTVNPF^360^	Envelope	4 (17%)	1,640 (1,050–1,870)
P7	^351^VGRLVTVNPFVATSSANSKV^370^	Envelope	19 (83%)	3,380 (2,720–3,880)
P8	^53^LAEVRSYCYHASVTDISTVA^72^	Envelope	10 (43%)	1,840 (1,300–3,370)
P9	^77^TGEAHNKKRADSSYVCKQG^95^	Envelope	3 (13%)	1,340 (1,020–1,660)
P10	^194^SGLNTEAFYVMTVGSKSFLV^213^	Envelope	2 (9%)	1,440 (930–1,750)
P11	^261^GLHQALAGAIVVEYSSSVKL^280^	Envelope	14 (61%)	3,060 (1,810–3,360)
P12	^471^MGVNARDRSIALAFLATGGV^490^	Envelope	12 (52%)	2,910 (14,300–3,580)
P13	^481^ALAFLATGGVLVFLATNVHA^500^	Envelope	5 (22%)	1,680 (1,060–1,810)
P14	^121^LQIGVHGILNAAAIAWMIVR^140^	NS2A	13 (57%)	2,820 (1,310–3,720)
P16	^108^NESSIMWLASLAIVTACAG^126^	Capsid	9 (39%)	1,540 (990–3,360)
P18	^73^SSQAGSLFVLPRGVPFTDLD^92^	NS4B	9 (39%)	1,830 (1,210– 2,950)
P19	^11^ADLKSMFAGKTQASGLTGLP^33^	NS4B	9 (39%)	1,880 (1,560–3,270)
P20	^21^TQASGLTGLPSMALDLRPAT^40^	NS4B	18 (78%)	3,170 (2,160–3,710)
P32	^100^KQNKRGGNEGSIMWLACLAV^119^	Capsid	18 (78%)	3,130 (2,210–3,550)
P33	^105^QITLTTFLTAMVLATLHYGY^124^	NS4B	(57%)	2,510 (1,610–3,410)
P34	^111^AAFFQLASADLQIGVHGILN^130^	NS2A	20 (87%)	3,430 (3,220–3,660)

### *Ex vivo* IFNγ ELISpot Responses to JEV Specific Peptides

Based on the results of cultured ELISpot responses, 18/23 (78.3%) of DENV^−^JEV^+^ individuals responded to 6/20 peptides tested and we wished to determine their immunogenicity using *ex vivo* ELISpot assays as we sought to investigate if the frequency of JEV-peptide specific T cells were present at frequency that they can be detected *ex vivo*.

**Figure 2:**
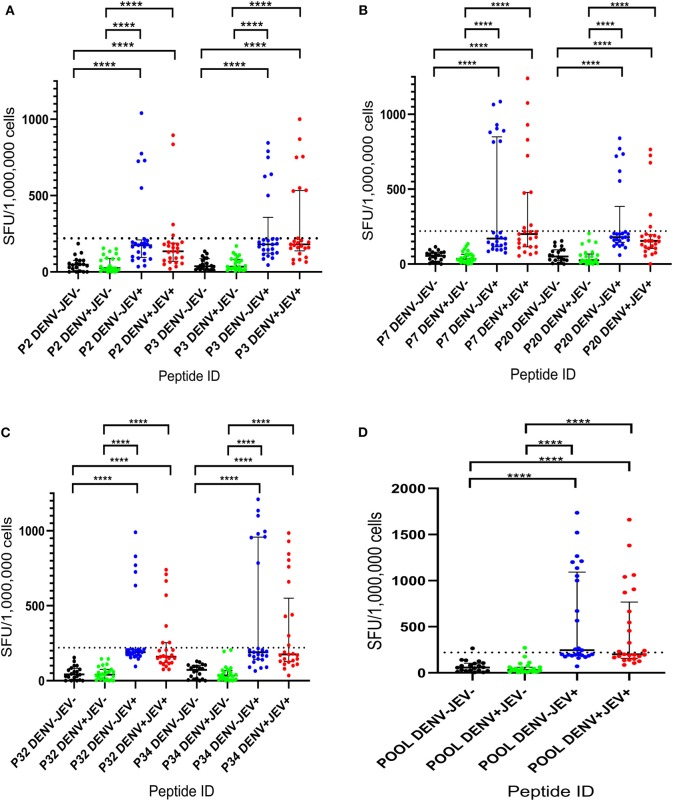
*Ex vivo* T cell immune responses to six JEV-specific peptides in individuals with varied DENV and JEV seropositivity. JEV-specific T cell responses were measured by ELISpot assay to 6 JEV-specific peptides (which were given higher T cell immune responses in culture ELISpot assays) in those who were both JEV and DENV seropositive (JEV^+^DEV^+^, *n* = 25), JEV seropositive but seronegative for DENV (JEV^+^DEV^−^, *n* = 25), DENV seropositive but JEV-seronegative (JEV^−^DENV^+^, *n* = 25), and seronegative for both (JEV^−^DENV^−^, *n* = 20). Error bars indicate the median and the interquartile range. The horizontal dotted line represents the cut-off value of 220 SPU/10^6^, which was considered as the mean, ± 3SD of the negative controls given spot count for all four groups. ^*^P < 0.05, ^**^P < 0.01, ^***^P < 0.001, ^****^P < 0.0001. The background has been subtracted from the responses displayed. Black color- JEV^−^DENV^−^; Green color- JEV^−^DENV^+^; Blue color- JEV^+^DEV^−^; Red color- JEV^+^DEV^+^. **(A)** *Ex vivo* T cell immune responses of P2 and P3. **(B)** *Ex vivo* T cell immune responses of P7 and P20. **(C)** *Ex vivo* T cell immune responses of P32 and P34. **(D)** *Ex vivo* T cell immune responses of pool.

*Ex vivo* T cell responses to the six 20mer peptides (P2, P3, P7, P20, P32, and P34) were evaluated in a second cohort of JE vaccinated, DENV seronegative individuals (JEV^+^DENV^−^, *n* = 25), JEV non-vaccinated (JEV seronegative) but DENV seropositive individuals (JEV^−^DENV^+^, *n* = 25), JEV vaccinated, DENV seropositive individuals (JEV^+^DENV^−^, *n* = 25), and both DENV and JEV seronegative (JEV^−^DENV^−^, *n* = 20). We also pooled all these peptides together and evaluated the *ex vivo* IFNγ ELISpot responses to this pool of peptides in the above 4 groups. The cutoff value for a positive IFNγ T cell response was considered as the mean ± 3SD of all negative controls, and in this study, it was 220 SFU/10^6^ PBMCs. As seen with the cultured ELISpot assays, none of the JEV^−^DENV^−^ and JEV^−^DENV^+^ individuals responded to any of the six JEV-specific peptides (Figure 2). The number of individuals who responded to each of the peptides is shown in Table 4. Again, quite a few individuals responded to these peptides, which were below the cut off value stimulated by us. However, we wished to have stringent assessment criteria to only select the T cell responses which displayed a high magnitude, so that false positive responses are not selected.

**Table 4:** *Ex vivo* IFNγ ELISPot responses to six of the immunodominant JEV-specific peptides.

**Peptide ID**	**Peptide sequence**	**Protein**	**Number of individuals who responded**		**Median (IQR) SFU/10**^****6****^		**CD107a expression (%)**	
			**DENV^**−**^JEV^**+**^ (*n* = 25)**	**DENV^**+**^JEV^**+**^ (*n* = 25)**	**DENV^**−**^JEV^**+**^**	**DENV^**+**^JEV^**+**^**	**CD4**	**CD8**
P2	^155^YSAQVGASQAAKFTVTPNAP^174^	Envelope	5 (20%)	4 (16%)	175 (95–210)	135 (70–189)	4.21	2.46
P3	^149^SENHGNYSAQVGASQAAKFT^168^	Envelope	6 (24%)	7 (28%)	180 (122.5–357.5)	180 (137.5–532.5)	-	-
P7	^351^VGRLVTVNPFVATSSANSKV^370^	Envelope	8 (32%)	9 (36%)	170 (115–850)	200 (115–477.5)	3.61	2.08
P20	^21^TQASGLTGLPSMALDLRPAT^40^	NS4B	6 (24%)	5 (20%)	180 (145–385)	155 (105–195)	2.55	1.44
P32	^100^KQNKRGGNEGSIMWLACLAV^119^	Capsid	5 (20%)	7 (28%)	190 (170–210)	160 (120–253.8)	-	-
P34	^111^AAFFQLASADLQIGVHGILN^130^	NS2A	8 (32%)	10 (40%)	190 (142.5–957.5)	175 (127.5–550)	-	-

Although none of DENV^+^JEV^−^ or DENV^−^JEV^−^ individuals responded to any of these peptides, in the *ex vivo* IFNγ ELISpot assays, the number of individuals of DENV-JEV+ and DENV^+^JEV^+^ groups who responded were also low. For instance, only 5 (20%)−10 (40%) individuals of each of the two groups responded any of these peptides *ex vivo*. Again, the most immunogenic peptide was P34, with 8–10 (32–40%) individuals responding to it.

### Investigating if JEV-Peptide Specific T Cell Responses Were of the CD4 ± or the CD8 ± Subtype

Following identification of six JEV-specific, highly conserved peptides, we further proceeded to determine if the T cells recognizing these peptides were predominantly CD4+ or CD8+ T cell subtype. As we determined the IFNγ-producing capacity using *ex vivo* and cultured ELISpot assays, in ICS assays we instead determined the degranulating capacity by assessing CD107a/CD4/CD8 expression *ex vivo* when stimulated with these peptides. In order to carry out these assays we re-recruited 4 individuals who were DENV^+^JEV^+^ and were found to respond to these peptides. We found that in these individuals, peptide 2, 7, and 20 were predominantly recognized by CD4+ T cells whereas the subset responding to peptide 34 was inconclusive. The CD107a expression to these peptides in these 4 individuals varied from 1.4 to 4.21% of the proportion of the CD4+ T cells. Very low CD1017a expression was induced by peptide 3 and 32 such that it was difficult to determine if the responding cells were CD4+ or CD8+. The gating strategy and an example CD107a expression for a JEV specific peptide is shown in Supplementary Figure 3.

As mentioned above, P34 was the most immunogenic JEV-specific peptide, recognized by 86.9% individuals who were DENV^−^JEV^+^, P3 and P7 were recognized by 82.6% of DENV^−^JEV^+^ group of individuals and P2, P32, and P20 recognized by 78.3% individuals. Apart from P34, P3, and P32, which the T cell subtype could not be determined, all other peptides were recognized by CD4+ T cells, thus likely presented by MHC Class II molecules. Although, we did not HLA type the 66 donors, we used the IEDB analysis resource to predict binding of these peptides to MHC class II alleles (36). The most immunodominant peptide P34 gave very high binding scores for many DQB1 alleles suggesting that it was likely to be presented by many different alleles (Supplementary Materials). For instance, it gave extremely high binding scores to different alleles of DQB1^*^02, DQB1^*^03, DQB^*^05, and DQB1^*^06 which are present in the 17.6, 20.6, 28.15, and 29.4%, respectively (37). The other JEV-specific peptides were also shown to bind to multiple MHC class II alleles, although at a lower frequency than P34 (Supplementary Materials).

## Discussion

In this study, we have identified highly conserved regions, specific to JEV which are recognized by JEV-specific T cells and were not recognized by any of DENV seropositive individuals. Identification of JEV-specific T cells that do not cross react with the T cells specific to the DENV, would be important to further understand the protective or pathogenic role of JEV specific T cells in acute JE infection and to find out how sequential infection with the DENV would affect the development of JEV-specific T cell immunity on subsequent exposure.

Of the six JEV-specific peptides which gave a high frequency of responses, three of the serotype specific regions identified were within the envelope of JEV (peptide 2, 3, and 7), and the other three regions were located in capsid (peptide 32), NS4B (peptide 20) and NS2a (peptide 34). T cell responses to these peptides were assessed in individuals who had received the JE vaccine and not in those who were naturally infected with the virus, as the JE vaccine was included in the National Immunization schedule in Sri Lanka since 1988, in a stepwise manner (38). Therefore, the incidence of natural JEV infection has been very low during the past two decades in Sri Lanka and it was not possible to find individuals naturally infected with the JEV recently. The live JE vaccine has shown to induce highly cross-reactive CD4+ and CD8+ T cell responses, which cross-react with DENV, and predominantly targeted the PrM, NS1 and NS3 regions (39). These regions, which are preferentially targeted by T cells following JE immunization, show a high degree of homology with DENV and many other flaviviruses (39).

The peptides identified within the envelope region had <35% homology with the envelope proteins of all DENV serotypes and the other peptides had 25% homology with the regions of all DENV serotypes. Peptide 34, which was the most immunogenic peptide recognized by 86.9% of individuals who had received the JE vaccine (and none of DENV immune individuals) showed <20% homology with any of DENV serotypes and 25% homology with yellow fever virus and Zika virus. Infection with either Zika or yellow fever virus has not been reported in Sri Lanka so far. However, sporadic cases of West Nile virus (WNV) have been reported (3, 40) and JEV-peptide 34 gives a 40% homology with the WNV, which could induce cross-reactive T cells. Two of the peptides (peptide 2 and 3) which were identified within envelope region of JEV and which did not induce any responses in DENV seropositive individuals, showed 55% homology with WNV. Peptide 7, again within the envelope region of JEV, had a homology of 75%, which may induce WNV cross-reactive T cell responses due to the degree of homology.

Although recognition of antigens by T cells is HLA-restricted and therefore, responses to these JEV specific peptides would depend on an individual's HLA type, we wished to identify responses that are recognized by a large proportion of JEV immune individuals irrespective of their HLA type. For instance, in acute DENV infection, although recent studies show that DENV-specific T cells are likely to be protective (9, 12, 15, 41), studies have also shown that DENV-specific T cells are highly cross reactive and possibly contribute to disease pathogenesis by producing pro-inflammatory cytokines (29, 42). However, identification of JEV-specific T cell epitopes that do not cross react with DENV, would also enable us to better understand T cell immunity to the DENV, in the context of background immunity to the JEV, especially following vaccination. Furthermore, it would also be useful to investigate if the magnitude and the phenotype of JEV-specific T cell responses influence the strength and breadth of the DENV-specific T cell response following natural infection or following immunization with the DENV. Therefore, we wished to identify JEV-specific T cell epitopes that could be used for this purpose. Knowing the HLA restriction of these epitopes would be important to characterize the phenotype of these T cells. Such experiments were beyond the scope of this study. However, since the JEV-specific epitopes identified in this study were not investigated in relation to the donor HLA types, these findings are broadly relevant to the population studied.

The responding T cell subset was not clear for peptide 34, 32, and 3, while responses to peptide 2, 7, and 20 were predominantly from CD4+ T cells. The low frequency of CD107a expression from P34, P32, and P3 was probably due to them being predominantly been recognized by CD4+ T cells too, which have a poor degranulation capacity. Furthermore, although there was detectable CD107a expression for peptide 2, 7, and 20 the responses were of relatively low frequency (between 1.4 and 4.2%), suggesting that these JEV-peptide specific T cells have overall poor degranulation capacity. However, in these assays we measured the capacity of these JEV-peptide specific T cells to degranulate and it is possible that IFNγ production could be by a completely different subset of T cells. The dominance of CD4+ T cell epitopes to JEV specific peptides, could be due to several reasons. Firstly, we assessed JEV-specific T cell responses in those who received the JE vaccine and not those who were naturally exposed. It was shown that those who received the JE vaccine are more likely to have a higher frequency of a CD4+ T cell response compared to those who were naturally infected with JEV (39). Secondly, we used cultured ELISpot responses to identify JEV-specific memory T cell responses. Although cultured ELISpot responses are a valuable tool in investigating memory T cell responses, it has been shown this process results in reduced proliferation of CD8+ T cells compared to CD4+ T cells (34). Therefore, our approach would have biased the memory JEV-specific responses toward finding memory CD4+ T cell responses. The high frequency of recognition of these peptides could also be due to their presentation by MHC class II molecules. For instance, 78% responded to peptide 2 while 83% responded to peptide 7. The CD107a expression for peptide 34 was generated by both CD4+ and CD8+ T cells. Epitopes presented by MHC class II alleles are shown to be highly promiscuous. The same epitope has shown to be presented by multiple T cell alleles in breast cancer (HER2) (43), Ag85B T-cell epitope in Mycobacterium tuberculosis (44), T cell epitopes in Mycobacterium leprae (45) and in many other instances.

Although we found that >75% of individuals responded to these 6 peptides by cultured ELIspot assays, the responses detected by *ex vivo* IFNγ ELISpot assays were less frequent. For instance, only 40% of individuals responded to peptide 34, by *ex vivo* assays, whereas 86.9% of individuals responded in the cultured ELISpot assays. Again, since the frequency of virus specific CD4+ T cells are known to be lower than the frequency of virus specific CD8+ T cells, is likely to be the reason for the limited responses detected by us through *ex vivo* ELISpot assays. In addition, as individuals with certain HLA types are only likely to present these peptides, these epitopes might not be presented by the individuals who showed negative responses. However, due to the low frequency of responses to these peptides *ex vivo*, the use of these peptides to evaluate JEV-specific T cell responses in a community or as a diagnostic test would not be suitable. Although a high frequency of responses was seen in cultured ELISpot assays, such assays would not be practical to be used as a diagnostic assay as they are labor intensive and expensive. Currently, one of the major challenges is distinguishing JEV or DENV T cell responses, when individuals are immune to both viruses. Since we have identified several JEV-specific peptides, they could be used to further understand the pathogenic or protective role of JEV-specific T cell responses.

One of the limitations of our study is the use of the JE Inbios IgG ELISA to define JEV-specific seropositivity. It has been shown that this assay has poor sensitivity and detected the presence of JEV specific antibodies in only 20% of those who were found to have JEV specific IgG by neutralization assays (46). In order to recruit JEV seronegatives, we only recruited those who had never received the JEV vaccine and none of the JEV^−^DENV^−^ responded to any of the peptides. Furthermore, we had a very high cutoff value in our cultured ELISpot assay, so that we only pickup responses of high magnitude so that low frequency possible non-specific responses are not taken into account.

In summary, both JEV and DENV co-circulate in the same regions and since JEV and DENV vaccines are likely to be co-administered in the same geographical regions in future. We had previously identified DENV serotype-specific T cell epitopes in conserved regions of all four DENV serotypes (24). Therefore, identification of these JEV-specific, conserved, immunogenic regions are likely to help in understanding T cell responses to both JEV and DENV independently of each other.

## Data Availability Statement

All datasets generated for this study are included in the article/Supplementary Material.

## Ethics Statement

The studies involving human participants were reviewed and approved by Ethics Review Committee of the University of Sri Jayewardenapura. Written informed consent to participate in this study was provided by the participants and their legal guardian/next of kin.

## Author Contributions

PP carried out the bioinformatics analysis, cultured, and *ex vivo* ELISpot assays. CJ recruited all individuals to the study and carried out the JEV ELISA. AW helped with both the cultured and *ex vivo* ELISpot assays and ICS assays. LG helped with the DENV and JEV ELISA. GO helped in designing the study and writing the paper. CG helped in the bioinformatic analysis, planning the study, and obtaining funding. GM helped in designing the study, data analysis, obtaining funding, and writing the manuscript.

### Conflict of Interest

The authors declare that the research was conducted in the absence of any commercial or financial relationships that could be construed as a potential conflict of interest.

## Abbreviations

JEV: Japanese encephalitis virus
DENV: Dengue virus
WNV: West nile virus.
