# Supplementary material for: Identification of Immune Responses to Japanese Encephalitis Virus Specific T Cell Epitopes
Source: Front Public Health. 2020 Feb 12;8:19. doi: 10.3389/fpubh.2020.00019 (PMC7029616; doi:10.3389/fpubh.2020.00019)
Supplement: Supplementary file 1 [file Data_Sheet_1.pdf]

1_V1

Supplementary Table 1: Allele prediction of peptide 34

Allel e	Amino acid position		Adjusted rank
	start	End	
DQB1* 0201	1	15	0.92
	2	16	1.70
	3	17	3.60
DQB1* 0202	1	15	0.92
	2	16	1.70
	3	17	3.60
DQB1* 0203	1	15	0.55
	2	16	0.99
	3	17	2.70
DQB1* 0204	1	15	0.92
	2	16	1.70
	3	17	3.60
DQB1* 0205	1	15	0.48
	2	16	0.89
	3	17	2.50
DQB1* 0206	1	15	0.92
	2	16	1.70
	3	17	3.60
DQB1*0301 to DQB1* 0338	1	15	0.8 - 5.0
	2	16	2.0 - 7.0
DQB1*0501 to DQB1*0514	1	15	3.30 to 6.0
DQB1*0601 to DQB1*0644	1	15	0.81 - 3.0
	2	16	2.50 – 6.0

Peptide

Allel e	Amino acid position		Scor e
	Start	End	
DQB1*0201 to DQB1*0206	6	20	4.0 – 7.0
DQB1*0301 to DQB1*0338	6	20	0.87 – 3.5
	5	19	0.88 – 5.0
DQB1*0501 to DQB1*0514	6	20	3.5 – 7.0
DQB1*0601 to DQB1*0644	5	19	0.12 – 3.5
	6	20	0.19 – 4.5

Peptide 34 (lower values in the adjusted rank, are predictive of a high binding strength (Vita et al., 2019)

Peptide 20

Allel e	Amino acid position		Scor e
	Start	End	
DQB1*0201 to DQB1*0206	3	17	4.8 – 9.5
DQB1* 0301 to DQB1*0338	2	16	0.98 – 5.0
	3	17	1.0 – 6.0
DQB1*0501 to DQB1*0514	5	19	4.5 – 10.0
DQB1*0601 to DQB1*0644	2	16	1.3 – 4.5
	3	17	1.7 – 5.0

Peptide 7

Allel e	Amino acid		Scor e
	Start	End	
DQB1*0201 to DQB1* 0206	1	15	3.5 – 8.0
DQB1*0301 to DQB1*0338	6	20	0.87 – 4.5
	5	19	0.93 – 6.0
DQB1*0501 to DQB1*0514	1	15	2.5 – 4.5

Peptide

	2	16	3.6 – 6.0
DQB1*0601 to DQB1*0644	2	16	0.71 – 4.0
	4	18	0.77 – 5.0

Peptide

Allel e	Amino acid		Scor e
	Start	End	
DQB1*0201 to DQB1*0206	5	19	5.6 – 9.0
DQB1*0301 to DQB1*0338	5	19	0.66 – 4.0
	6	20	0.83 – 4.5
DQB1*0 01 to DQB1*0514	5	19	4.8 – 11.0
DQB1*0601 to DQB1*0644	5	19	0.76 – 4.5
	6	20	0.82 – 5.0

Peptide 2

Allel e	Amino acid		Scor e
	Start	End	
DQB1*0201 to DQB1*0206	1	15	4.8 – 11.0
DQB1*0301 to DQB1*0338	1	15	0.92 – 4.0
	2	16	0.95 – 5.0
DQB1*0501 to DQB1*0514	1	15	4.6 – 12.0
DQB1*0601 to DQB1*0644	1	15	0.88 – 4.5
	2	16	0.92 – 6.0

References

Vita, R., Mahajan, S., Overton, J.A., Dhanda, S.K., Martini, S., Cantrell, J.R., et al. (2019). The Immune Epitope Database (IEDB): 2018 update. *Nucleic Acids Res* 47(D1)**,** D339-D343. doi: 10.1093/nar/gky1006.
