# Supplementary material for: Identification of Immune Responses to Japanese Encephalitis Virus Specific T Cell Epitopes
Source: Front Public Health. 2020 Feb 12;8:19. doi: 10.3389/fpubh.2020.00019 (PMC7029616; doi:10.3389/fpubh.2020.00019)
Supplement: Supplementary file 2 [file Data_Sheet_2.pdf]

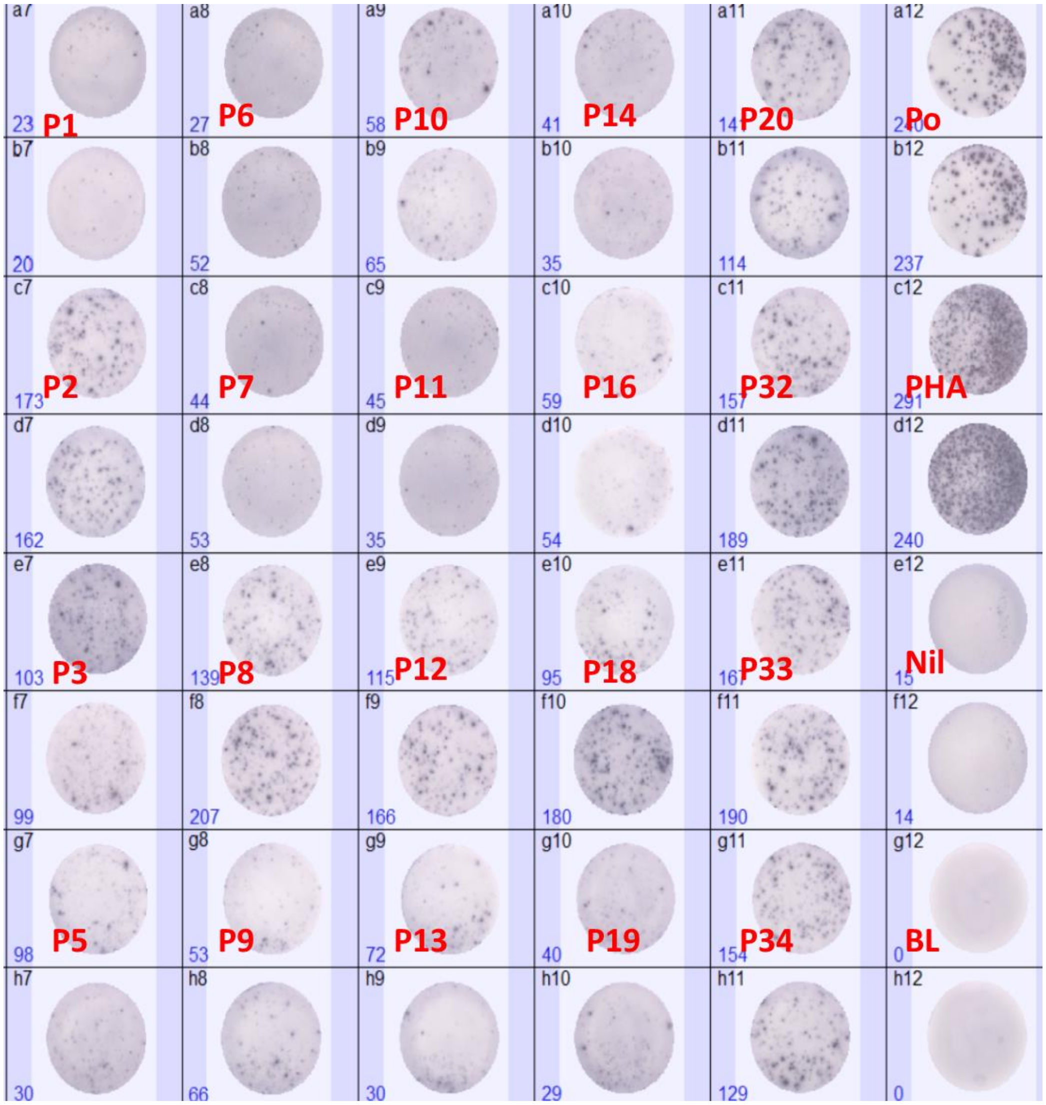

Supplementary figure 1: An example of an cultured ELISpot assays, outlining the plate set up and responses to each of the 20 peptides and the positive control (PHA) and the negative control (Nil)
