# Supplementary figures and images for: Identification of Immune Responses to Japanese Encephalitis Virus Specific T Cell Epitopes
Source: Front Public Health. 2020 Feb 12;8:19. doi: 10.3389/fpubh.2020.00019 (PMC7029616; doi:10.3389/fpubh.2020.00019)

3\_V1

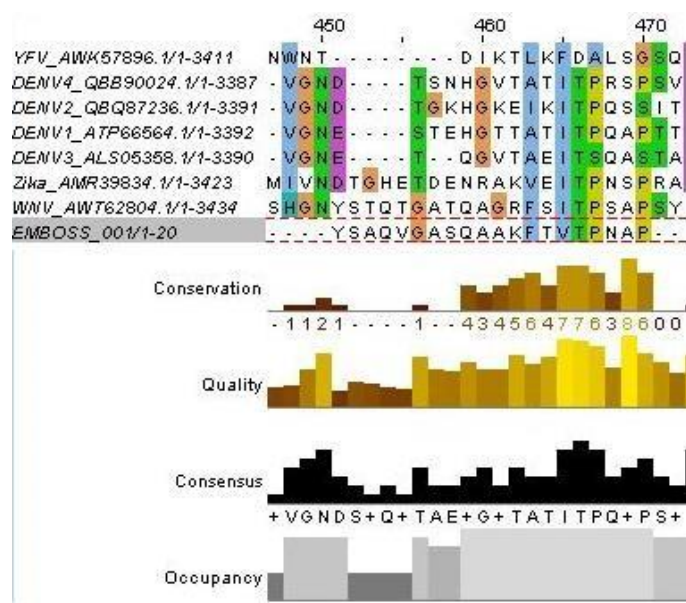

Peptide 2

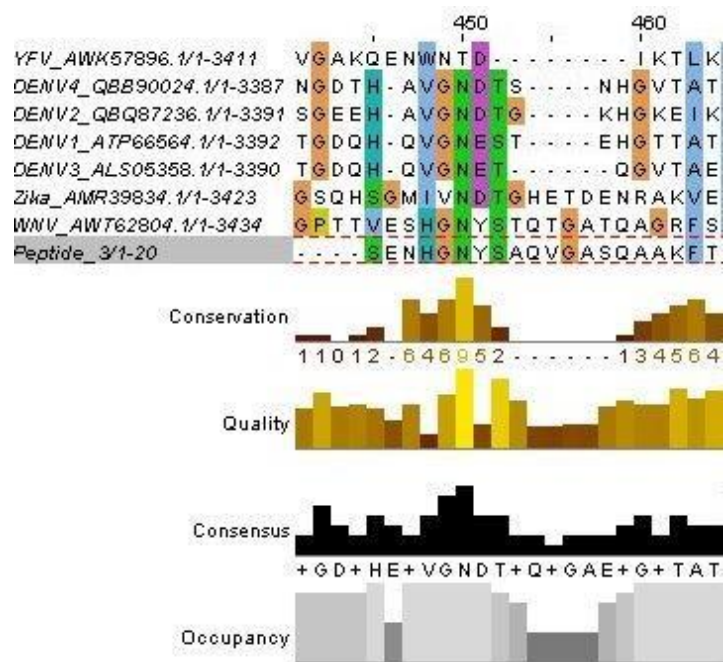

Peptide

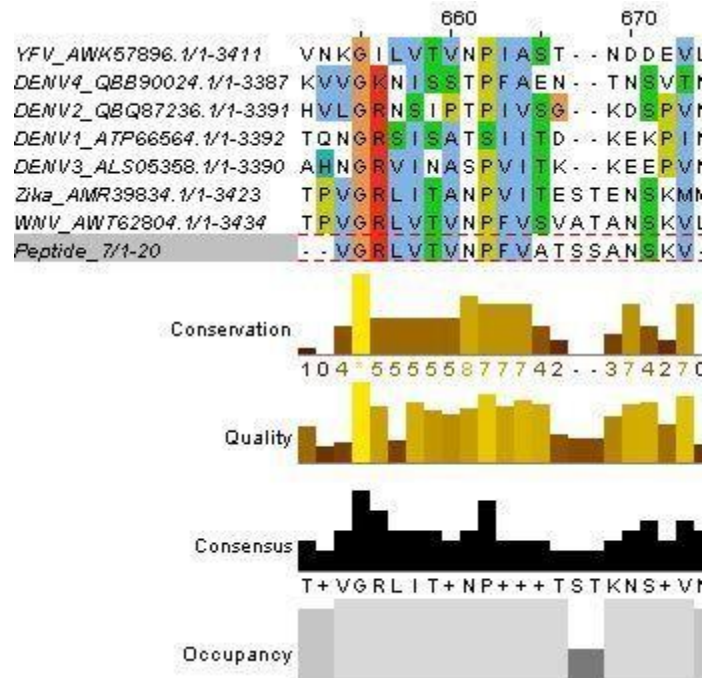

### Peptide 7

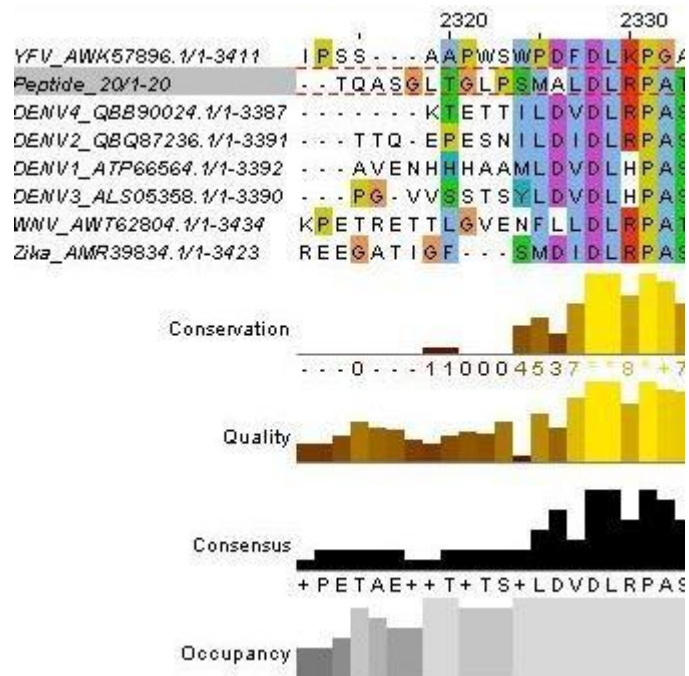

### Peptide

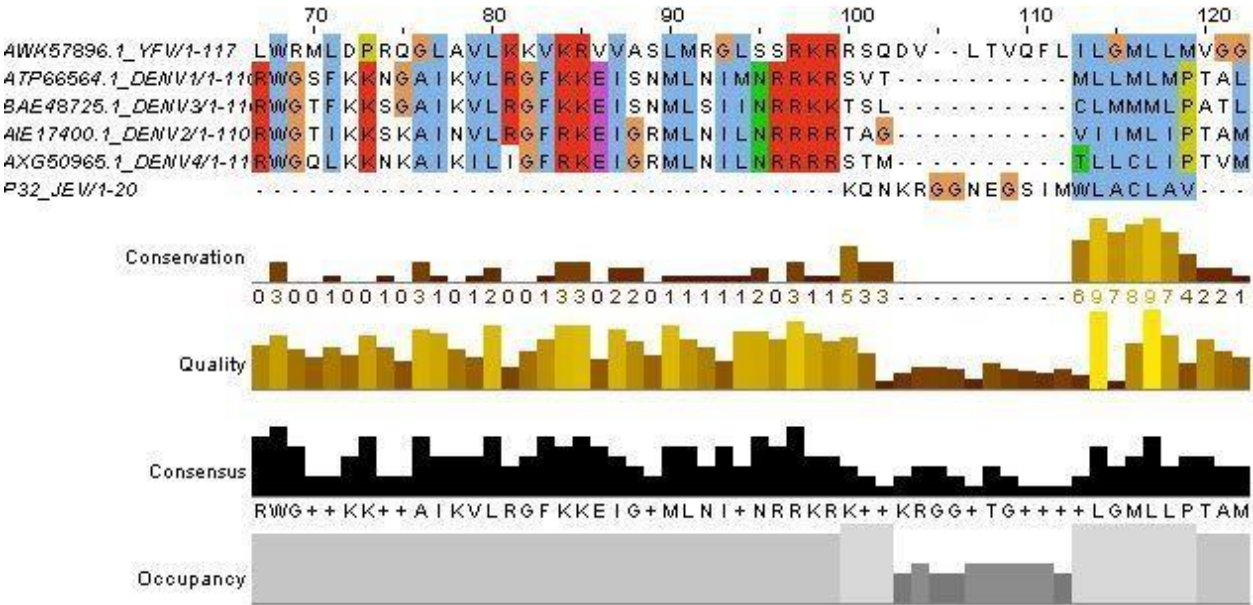

Peptide 32

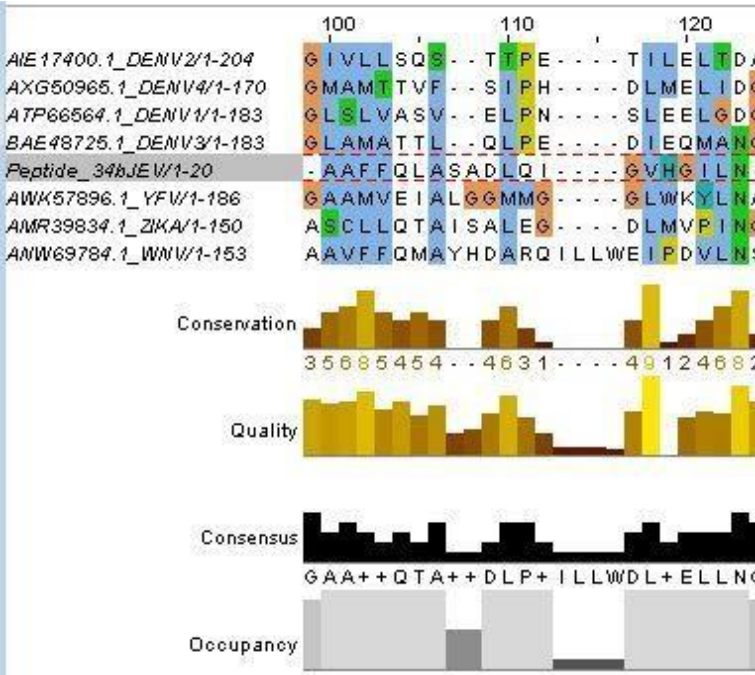

Supplementary figure 2

Peptide

Supplement: Supplementary file 3 [file Data_Sheet_3.pdf]
