# Supplementary material for: Identification of Immune Responses to Japanese Encephalitis Virus Specific T Cell Epitopes
Source: Front Public Health. 2020 Feb 12;8:19. doi: 10.3389/fpubh.2020.00019 (PMC7029616; doi:10.3389/fpubh.2020.00019)
Supplement: Supplementary file 4 [file Data_Sheet_4.pdf]

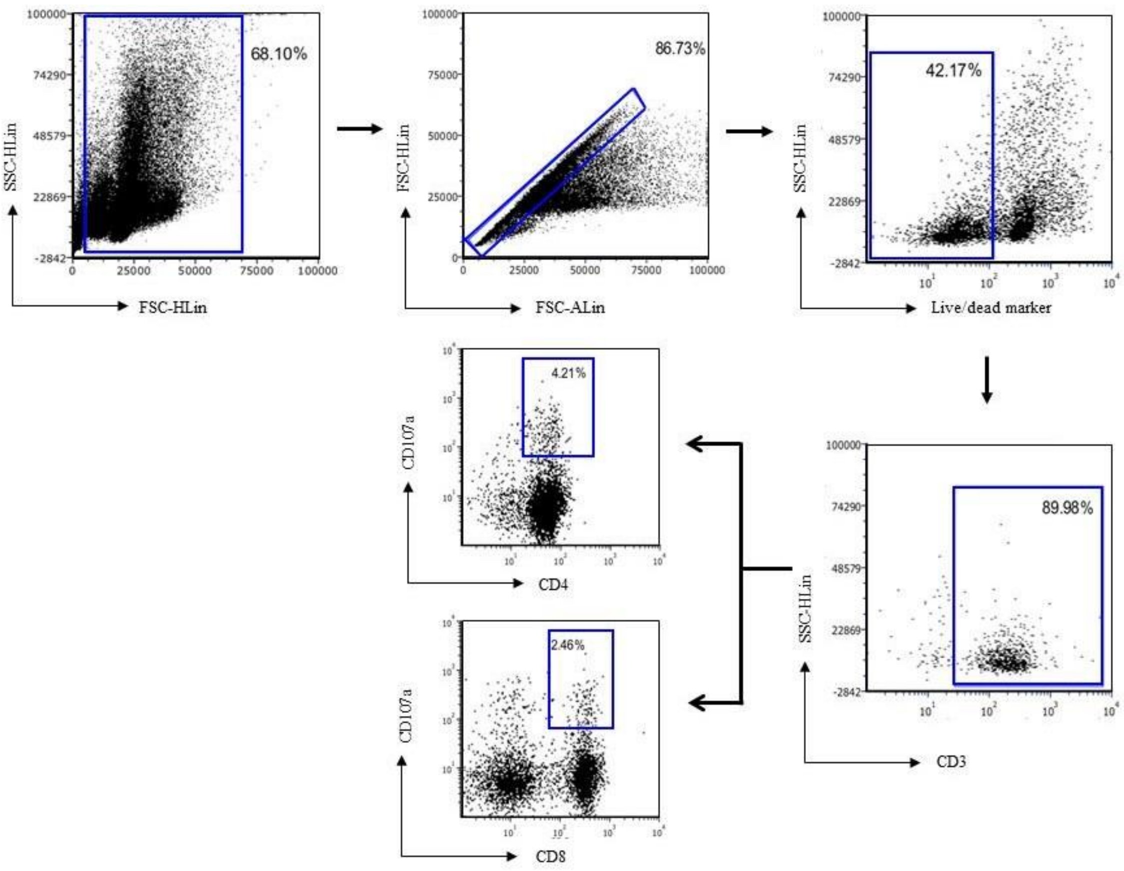

4_V1. Supplementary figure 3: The gating strategy in identifying CD107a expression by CD4+ and CD8+ T cells specific to the JEV peptides.
